# Blood-based biomarkers in pancreatic ductal adenocarcinoma: developments over the last decade and what holds for the future- a review

**DOI:** 10.3389/fonc.2025.1555963

**Published:** 2025-04-22

**Authors:** Ronit Juthani, Ashish Manne

**Affiliations:** ^1^ Department of Medicine, Saint Vincent Hospital, Worcester, MA, United States; ^2^ Department of Internal Medicine, Division of Medical Oncology at the Arthur G. James Cancer Hospital and Richard J. Solove Research Institute, The Ohio State University Comprehensive Cancer Center, Columbus, OH, United States

**Keywords:** pancreatic ductal adenocarcinoma, biomarkers, blood, DNA, RNA, protein

## Abstract

Pancreatic Ductal Adenocarcinoma (PDAC) accounts for a significant burden of global cancer deaths worldwide. The dismal outcomes associated with PDAC can be overcome by detecting the disease early and developing tools that predict response to treatment, allowing the selection of the most optimal treatment. Over the last couple of years, significant progress has been made in the development of novel biomarkers that aid in diagnosis, prognosis, treatment selection, and monitoring response. Blood-based biomarkers offer an alternative to tissue-based diagnosis and offer immense potential in managing PDAC. In this review, we have discussed the advances in blood-based biomarkers in PDAC, such as DNA (mutations and methylations), RNA, protein biomarkers and circulating tumor cells (CTC) over the last decade and also elucidated all aspects of practical implementation of these biomarkers in clinical practice. We have also discussed implementing multiomics utilizing more than one biomarker and targeted therapies that have been developed using these biomarkers.

## Introduction

Pancreatic ductal adenocarcinoma (PDAC) accounts for around 3% of all cancers in the United States and about 7% of all cancer deaths estimated in 2023 (1). The last few years have seen a significant increase in the global burden of PDAC diagnosis. The American Cancer Society projects that in 2025, approximately 67,440 patients will be diagnosed with PDAC, and an estimated 51,980 will succumb to the disease (2). Males are reported to have a higher prevalence of PDAC compared to females. While smoking rates have declined over recent decades, the rising incidence of diabetes is hypothesized to be a key driver of the increasing PDAC cases. Biomarkers such as fasting blood glucose and insulin resistance have shown significant associations with PDAC, and patients with the disease frequently present with elevated HbA1c levels (3). In common clinical practice, PDAC is classified into resectable, unresectable and borderline resectable to determine the possibility of surgical intervention and the need for neoadjuvant therapy (4, 5). A large proportion of patients have unresectable disease at the time of diagnosis (6). At the time of initial presentation, 50% patients are found to have metastatic PDAC, with 30-35% being unresectable and only 10-15% being amenable to surgery (3). There may also be the presence of pancreatic intraepithelial neoplasms (PanIN) which may progress to high-grade dysplasia and PDAC (3).

Early-stage PDAC is clinically silent while patients with advanced disease have nonspecific signs and symptoms in the form of anorexia, weight loss, abdominal pain, jaundice, acholic stools, and dark urine which are the manifestations of biliary tract obstruction. The most commonly used diagnostic tools include tri-phasic pancreatic protocol computed tomography (CT), magnetic resonance imaging (MRI), and endoscopic ultrasound-guided fine needle aspiration for cytological diagnosis (7). Serum carbohydrate antigen (CA 19-9) has been used to aid diagnosis in symptomatic patients and to predict recurrence after resection but its success as a screening tool has been underwhelming. There has been no effective strategy so far to screen and detect PDAC in early stages.

The management for PDAC currently depends on conventional polychemotherapies with poor outcomes and targeted therapy are seldom used (8). The dismal outcomes associated with PDAC necessitate the urgent need to develop tools that can identify cancer early (9). There is also an urgent need for tools to predict prognosis and response to treatment. There has been a huge push in the last couple of years towards the development of novel serum biomarkers that can aid in diagnosis, prognosis and assist in tailoring treatment and monitoring response post-treatment.

Based on current practice, tissue biopsies are the current method to assess molecular information about the tumor and essential for diagnosis, screening, and mutation expression. Tissue biopsies come with its own inherent set of disadvantages: the requirement of invasive surgery, which poses risks for the patient (10). Besides, certain tumors are difficult to access on account of challenging anatomical locations, including in PDAC. There is also an associated risk of augmenting the risk of metastatic seeding (11). There can also be variations in the amount of tissue extracted and results obtained due to the tumor heterogeneity, inter-operator variation, and evolving nature of the tumor. Lastly, the costs involved and the requirement of an operation theatre can be an inhibiting factor that is a hindrance to tumor characterization (12). Tumor monitoring is required at different times of the disease course to monitor treatment, and it is not feasible to have repeat biopsies every time (13). Monitoring using radiology while providing a good physical estimation hardly provides any evidence of molecular/pathological features that are vital to understanding the prognosis and treatment (14). Liquid biopsy and blood-based biomarkers are based on the principle that during apoptosis and necrosis, a few of these biomarkers are released into circulation, and their identification and capture provide an accurate molecular and pathological characterization of the tumor (15). The non-invasive nature of this process is appealing both to the patients and the clinician to avoid potential complications and morbidity associated with invasive biopsy measures and allow for dynamic monitoring of the tumor at different time points (16). Studies have been conducted in several malignant tumors with utility such as prostate, colorectal, lung, and breast with a great amount of research also having been conducted on PDAC (17–20).

While samples for liquid biopsy can be derived from both plasma and serum, plasma samples are often preferable to serum samples for analysis in clinical applications. This stems from the fact that while serum may have higher amounts of circulating free DNA, RNA and proteins, it is confounded by the presence of DNA, RNA and proteins released during the lysis of circulating white blood cells, including neutrophils, that reduce the relative proportion of tumor DNA, RNA and protein (21). It is also a prerequisite to collect serum at room temperature for clotting, which further increases the risk of cell lyses and degradation of DNA, RNA and protein (22). Most studies, however, are based on serum samples in spite of the advantages of plasma in this regard. This is due to the convenience of serum use in clinical laboratories or because of limitations in sample availability for retrospective studies (22).

In our 2022 publication, we explored the potential of cell-free DNA (cfDNA) testing (methylation and mutation) in clinical settings (23). Despite its promising results in retrospective and limited prospective studies, no new clinical applications have emerged since then. In the sections that follow, we discuss other important blood-based biomarkers such as RNA, proteins, circulating tumor cells (CTC) and multiomic tests in addition to cfDNA. We have focused on the recent advances in blood-based biomarkers such as DNA (mutations and methylations), CTC, RNA, and protein, and all aspects of practical implementation of these biomarkers in clinical practice.

## Cell-free DNA

Cell-free DNA (cfDNA) encompasses normal cell DNA, tumor cell DNA, and exosomal DNA (24). As cancer cells undergo apoptosis or necrosis, genetic material is aberrantly released into the bloodstream as either free nucleic acid or exosomes (24). It has been hypothesized that cfDNA can potentially be used to diagnose and stage PDAC, and research is also being conducted to investigate the role of cfDNA as a potential predictor of treatment response. Multiple studies have demonstrated over the last couple of years the presence of high levels of cfDNA in blood of patients with invasive tumors including pancreas, colon, and melanoma (25–27). cfDNA in PDAC was first reported by Shapiro et al. in 1983 when he found that cfDNA was significantly elevated in PDAC compared to healthy controls (28). He hypothesized its utility as a potential diagnostic and prognostic biomarker. In the past decade, we have made huge strides in implementing cfDNA into clinical practice. This has been supported by multiple studies demonstrating the high degree of correlation that exists between tumor DNA and cfDNA (29).

Numerous techniques are available to detect cfDNA of tumor cells. The most popularly used techniques include droplet digital PCR (ddPCR), Whole Genome Sequencing (WGS) and Whole Exome Sequencing (WES) (30). Other techniques that have also been used in studies include Beads, Emulsion, Amplification, and magnetics (BEAMing), Cancer Personalized Profiling by Deep Sequencing (CAPP-Seq), Tagged-amplicon deep sequencing (Tam-Seq) (30). ddPCR identifies rare mutations and copy number variations. The major limitation in ddPCR, in spite of its high sensitivity, is that it is only able to detect specific genomic sequences in the sample (31). BEAMing, while combining PCR with flow cytometry, also suffers from similar limitations (32). CAPP-Seq uses large genomic libraries in tandem with individual patient sample sequences to identify cfDNA alterations. It compares well-known tumor alterations with DNA oligonucleotides to identify patient-specific alterations (33). While there is an advantage in the identification of insertions/deletions, single nucleotide variants, rearrangements, and copy variants, it often struggles to identify fusions, which is not a problem with ddPCR, WES, or WGS. Tam-Seq employs primers to tag and identify the desired genomic sequence but requires that the sequence be characterized to be included in the analysis (34). WGS and WES analyses all the potential tumor mutations present in the entire genome allowing for comprehensive analysis and characterization. The extensive coverage comes with its own downside related to the increased cost, error rate and sensitivity (35).

In our previous review (23), we have elucidated how cfDNA somatic mutations can either be divided into specific mutations or nonspecific total mutated cfDNA. Numerous studies in the past have implicated KRAS mutations as a poor prognostic biomarker in PDAC. It has been associated with advanced and metastatic diseases and many studies have evaluated and found the presence of mutated KRAS and its quantification as important factors in determining the prognosis of PDAC (36). Ankeny et al. (37) conducted one of the earlier studies to clinically explore the utility of mutated KRAS in circulating tumour DNA (ctDNA) and its implication in PDAC. Using techniques of immunocytochemistry and chip mounting, they counted the number of CTC in PDAC and healthy controls. 78% patients in the PDAC group were found to have CTC in peripheral venous blood compared to 3.6% in the non-adenocarcinoma group. There was also a statistically significant difference that existed between stage 4 and other PDAC. Overall CTC had an Area Under Curve (AUC)=0.885 for discriminating locoregional disease versus metastatic disease. Other frequently detected mutations in cfDNA of patients with PDAC besides KRAS include TP53 and CDKN2A, with ATM, PIK3CA, PTEN, TERT, NF1, JAK2, and GNAS mutations also seen (29, 38). In a previous paper from 2022, we have described in detail about the cfDNA genomic and epigenetic biomarkers that have been studied to aid in the diagnosis of PDAC and its role in determining the stage and prognosis in this population (23). We will look at the most recent advances (last 3 years) in the field in this section (**Table 1**).

**Table 1:** Latest developments in prognosis, and treatment response prediction of PDAC using DNA biomarkers.

Authors	Year	Target	Method used	Results
Diagnostic biomarker				
Herreros-Villaneuva (39)	2022	KRAS, BRCA2, FLT3, HNF1A	NGS	Pathogenic mutations were detected only in 50% plasma samples of patients with stage 3-4 PDAC.
Wang et al. (41)	2022	KRAS+ EUS-FNA	ddPCR	Adding KRAS mutation increased the sensitivity and specificity of EUS-FNA from 71.4% to 91.6% and from 75.8% to 88.6% respectively.
Sellahewa et al. (40)	2023	KRAS G12/13 mutation	ddPCR	ctDNA KRAS G12/13 mutations were detected in 63% of all patients with PDAC with a specificity and tissue concordance of 100%.
Prognostic biomarkers				
Umemoto et al. (44)	2023	KRAS mutations in ctDNA	NGS	KRAS mutation detection rate was higher with metastasis to the liver(78%). In addition, median maximum variant allele frequency (VAF) was higher with metastasis to liver(1.9%).
Shah et al. (47)	2024	KRAS ctDNA	NGS	In patients treated with neoadjuvant chemotherapy, the presence of KRAS ctDNA at diagnosis was associated with and independently predicted worse PFS.
Lim et al. (48)	2024	ATM, BRCA1, BRCA2, MLH1, KRAS	ddPCR	Patients with alterations in DNA-damage repair genes showed better PFS (26.6 months vs 13.5 months, p=0.004). Patients with KRAS mutations had worse OS (8.5 months vs not applicable, p=0.003).
Eckhoff et al. (49)	2024	16 individual-specific, somatic single nucleotide variants ctDNA	ddPCR	During the immediate postoperative period (up to 9 weeks post-surgery), PFS and OS were significantly inferior in patients who were ctDNA-positive versus ctDNA-negative (PFS 97 versus 297 days, p < 0.001; OS 110 versus 381 days, p < 0.001
Motobayashi et al. (52)	2024	KRAS-mutated ctDNA	ddPCR	An increase in KRAS-mutated ctDNA values(day 0-7) was found to be associated with significantly shorter PFS (HR-24.234, p=0.0002).
Huang et al. (53)	2024	KRAS, TP53, SMAD4, CDKN2A, ARID1A	ddPCR	Patients with high KRAS in ctDNA significantly more frequently had an OS(p<0.001) and PFS<6 months(p=0.027), high TP53(p<0.001), ARID1A(p=0.040), SMAD4(p=0.007) ctDNA OS<6 months, high CDKN2A ctDNA PFS<6 months(p=0.048)
Till et al (PRINCE trial) (54)	2024	ctKRAS G12D, G12V	ddPCR	Higher baseline G12D was strongly associated with worse OS (log-rank p=0.0010). Early-on therapy clearance of G12D strongly associates with OS(p=0.0002).
					Halkova et al. (55)	2024	KRAS ctDNA	ddPCR	Patients with G12D mutation had six times shorter survival compared to patients without mutation(27 days vs 161 days, p=0.02).
Maulat et al. (56)	2024	ctDNA, cfDNA	ddPCR	Intraoperative ctDNA detection in peripheral blood was associated with worse PFS(HR-3.26,p=0.010) and OS(HR-5.46, p=0.002).					
					Treatment response prediction				
Evrard et al. (43)	2022	KRAS mutated ctDNA on Day 0 and Day 28	ddPCR	Score combining cfDNA at diagnosis >= or <30 ng/mL and presence or not of KRAS-mutated ctDNA at day 28 was an optimal predictor of cDCR(OR=30.7), PFS(HR=6.79),OS(HR=9.98)
Kitahata et al. (45)	2023	KRAS postoperative ctDNA	ddPCR	Patients with postoperative positive ctDNA had significantly shorter median OS(723 days) than patients with negative ctDNA results(not reached). Combined ctDNA and CA19-9 showed cumulative effect on both PFS(p=0.0066) and OS(p=0.0046).
Hata et al. (46)	2023	KRAS postoperative ctDNA	ddPCR	Patients with detectable postoperative ctDNA showed worse DFS(p=0.034) and OS(p=0.022). Patients with postoperative ctDNA were more prone to developing hepatic recurrence than with undetectable postoperative ctDNA(p=0.039).
Edland et al. (50)	2023	KRAS ctDNA	ddPCR	Longitudinal ctDNA measurements during chemotherapy successfully revealed disease progression in 20 (67%) of 30 patients with ctDNA detected at baseline, with a median lead time of 23 days (P = 0.01) over radiological imaging.
Sudo et al. (51)	2024	BRCA1/2, ATM	NGS	Objective response and PFS on platinum-containing chemotherapy were significantly better in patients with germline BRCA1/2 mutations (63.2% vs 16.2% and HR=0.55, respectively)

ctDNA, Circulating tumor DNA; NGS, Next generation sequencing; PFS, Progression-free survival; ddPCR, digital droplet polymerase chain reaction; OS, Overall survival; cfDNA, Circulating-free DNA; DCR, Disease control rate.

Numerous studies have been conducted in the last 3 years utilizing cfDNA in diagnosing PDAC. Herreros-Villaneuva (39) performed next-generation sequencing (NGS) on the plasma samples of 27 patients with PDAC between 2016 and 2020 in Spain using a commercial panel of 65 genes to detect cfDNA but found that pathogenic mutations were detected only in 50% of samples from patients with stage 3-4 PDAC, highlighting the need for further work in this area. Sellahewa et al. (40) utilized ddPCR in 81 patients with PDAC and 30 patients with benign pancreatic disease to analyze for KRAS G12/G13 ctDNA mutation and found that KRAS G12/G13 mutations were detected in 63% of all patients with PDAC and in 76% of patients who also had the same mutation detected in primary. The specificity and tissue concordance were 100%. KRAS has also been used in conjunction with endoscopic ultrasound-guided fine-needle aspiration (EUS-FNA) by Wang et al. (41) who found that the addition of the ctDNA biomarker increased the sensitivity and accuracy of EUS-FNA from 71.4% to 91.6% and 75.8% to 88.6% respectively. This probably represents a more accurate role of ctDNA in PDAC diagnosis, where it can serve in conjunction with other invasive diagnostic techniques that otherwise perform poorly by themselves. Pol et al. (42) took a unique approach, collecting samples from 203 healthy and 664 cancer plasma detection encompassing 12 cancer types and employed whole genome sequencing to analyze the plasma mitochondrial DNA fraction. They found that the mitochondrial DNA fraction was elevated in 5 cancers-cholangiocarcinoma, colorectal, liver, pancreatic, and prostate cancer, in comparison to healthy individuals. This elevation in mitochondrial DNA was independent of the remaining cfDNA fraction. A predictive model integrating mitochondrial DNA and copy number analysis increased the AUC from 0.73 when using copy number alterations alone to 0.81.

An interesting study that was conducted post-2022 was the KRASCIPANC study by Evrard et al. (43) who collected serum samples from patients with unresectable PDAC on day 1 and before each cycle of chemotherapy at different time points until the progression of the disease. They developed an interesting risk stratification tool that helped better understand the prognosis by taking into account the ctDNA of mutated KRAS at day 0 and day 28 to classify PDAC into best, mid, and worst subcategories with disease control rates of 88%, 53%, and 20% respectively. This essentially described a study that serves a dual purpose of providing an understanding of the prognosis and treatment response. Another important study was the Gozila study by Umemoto et al. in Japan (44). Stratifying KRAS mutation rates by sites of metastasis, they found that detection of KRAS mutations in ctDNA was significantly higher in patients with metastasis to liver (75%) compared to lymph nodes (60%) and lungs (46%). The median maximum variable allele frequency (VAF) was also significantly higher with liver metastasis (1.8%) than other sites. Another notable study conducted by Kitahata et al. (45) found a strong correlation between the presence of postoperative ctDNA and overall survival (positive ctDNA with an OS of 723 days versus not reached for the absence of ctDNA). Findings of Hata et al. (46) were in agreement and they found that detection of postoperative ctDNA was associated with worse overall survival (OS) and progression-free survival (PFS). However, contrary to other studies, they concluded that preoperative ctDNA did not affect long-term outcomes. Shah et al. (47) investigated the utility of ctDNA in localized PDAC to guide clinical decisions. Using a 105 NGS gene panel to investigate patients with localized PDAC, they found that the presence of baseline ctDNA was associated with a worse CA19-9 response than those in which baseline ctDNA was not detected, thereby providing an alternative biomarker of treatment response to neoadjuvant chemotherapy (NAC). Lim et al. (48) investigated the prognostic value of DNA damage repair genes (ATM, BRCA1, BRCA2, MLH1) and found the presence of mutations in DNA damage repair genes were associated with a significantly better PFS compared to those without mutations in these genes (PFS 26.6 versus 13.5 months). A hopeful prospect for the future is the possibility of using individualised ctDNA panels for assessing the prognosis and treatment response as assessed by Eckhoff et al. (49) who tracked individual specific 16 single nucleotide variants for ctDNA detection and described that its presence was associated with worse RFS and OS. ctDNA represents a growing field in PDAC to predict the prognosis and treatment response. One study by Edland et al. (50) has also investigated how longitudinal ctDNA monitoring revealed disease progression in 67% patients with a median lead time of 23 days over radiological imaging. Other possible mutations such as germline BRCA1/2 mutations may demonstrate a better response to platinum containing chemotherapy and may prove a vital role in decision making (51).

The sensitivity of cfDNA also depends on the number of mutations and alterations identified, which can pose unique challenges. Detecting a single mutation in thousands of circulating cfDNA can significantly affect the ability to identify cancer. In contrast, the detection of a larger number of alterations in the genome increases the sensitivity by increasing the probability. This has also been demonstrated in Monte Carlo simulations that have shown that increasing the number of abnormalities from a few to hundreds can improve their detection (57). This forms the basis for fragmentomics, a novel approach developed over the last few years that is based on low coverage of WGS of isolated cfDNA. One of the first instances was described by Cristiano et al. (58) in 2019, who developed a novel technique called as DELFI (DNA evaluation of fragments for early interpretation) to analyze genome-wide differences in fragmentation patterns. Analyzing the fragmentation profiles of 236 patients with breast, colorectal, lung, ovarian, pancreatic, gastric, or bile duct cancer using machine learning, they found that incorporating genome-wide fragmentation features had sensitivities of detection ranging from 57% to >99% among the different cancers with a specificity of 98% and an overall AUC of 0.94 (58). Three primary surrogates can be measured in fragmentomics: the fragment size, the end motif, and the nucleosome imprint. Numerous techniques have been described that assist in the implementation of fragmentomics: calculation of tumor fraction by enriching short fragments, motif diversity score (MDS), orientation-aware cfDNA fragmentation (OCF), windowed protection score (WPS), DNA evaluation of fragments for early interception (DELFI), fragmentation evaluation of epigenetics from cfDNA (FREE-C), Epigenetic expression interference from cell-free DNA-sequencing (EPIC-seq) (59). The ideal choice of sample for fragmentomics analysis was determined to be plasma by Lee et al. (60), who found that while large fragments were increased in serum, the KRAS-mutated fraction in serum was significantly lower than that of plasma. One significant challenge in the implementation of large-scale fragmentomics is that the small amount of cfDNA makes library construction difficult, and the limited screening accuracy in different states limits its utility in cancer screening (59).

There have been numerous studies done specific to PDAC in fragmentomics. Liu et al. (61) utilized hybrid-capture-based cfDNA sequencing to analyze cfDNA fragments in PDAC and found that increasing the threshold of fragments decreases the ability to detect KRAS mutations in plasma, and the ability to recover ultra-short fragments increased the performance to detect KRAS mutations. This was followed up by Zvereva (62), who studied the plasma DNA samples of 40 patients with PDAC already known to carry KRAS mutation at codon 12 and screened cfDNA using a 4-size amplicon strategy to determine if their size would change detection rates, in turn giving an idea of malignant vs non-malignant cfDNA fragment sizes. They found that higher KRAS amplicon size (167bp and 218bp) was significantly associated with lower detectable cfDNA mutant allelic fractions (p<0.0001). One of the latest studies was performed by Shi et al. (63) who utilized fragmentomic features of pancreatobiliary cancers and combined them with CA19-9 to create a stacked model that was able to distinguish the respective cancers with an AUC of 0.978 in the training cohort and 0.941 in the validation cohort and performed fairly well even with low-coverage sequencing depth (AUC=0.905). Integrating with CA 19-9 enhanced the performance of the stacked model even further, achieving an AUC as high as 0.995.

Incorporating ctDNA into clinical practice with PDAC in supplementation with radiological imaging and its translation into better outcomes represents an exciting research proposition that needs further investigation.

### DNA methylation markers

DNA methylation is known to affect a variety of processes in cells, including imprinting, transcriptional regulation and integrity of developmental processes (64). It mediates its influence via its effects on promoters and enhancers (noncoding DNA elements), which play an important role in gene regulation. In a normal cell, it maintains genomic integrity by methylating the 5’ position of the cytosine ring through a family of enzymes called as DNA methyltransferases (DNMT). This occurs in regions of repetitive genomic regions, including satellite DNA, long interspersed transposable elements (LINEs) and short interspersed transposable elements (SINEs). The principle of inhibiting gene expression is secondary to two mechanisms-direct inhibition by inhibition of specific transcription factors or indirectly through recruitment of methyl-CpG-binding domain proteins (65). DNA methylation can contribute to cancer in three ways: first, by hypomethylation of the cancer genome disinhibiting tumorigenesis, second by focal hypermethylation at tumour suppressor regions, and third, by direct mutagenesis through deamination, UV irradiation or exposure to carcinogens (66).

There are three main techniques for identification of differentially-methylated regions- bisulfite conversion-based methods, restriction enzyme-based approaches and affinity enrichment-based assays (67). Bisulfite-based methods are utilized the most largely due to its economical nature and rapid results. On the other hand, restriction-based enzyme approaches, while being more accurate, are limited due to the higher costs involved in their implementation. The third method of using an enrichment-based assay is easy with good sensitivity and specificity. However, one limitation of this approach is enrichment of sequences with higher number of CpG is more frequent than CpG poor fragments leading to its underrepresentation (67).

DNA methylation profiles of malignant conditions can be undifferentiable from non-malignant conditions if they are derived from the same tissue. One of the first studies utilizing the methylation signature in cfDNA was performed by Leman-Werman (68), who were able to interrogate methylome databases to identify cell/tissue-specific methylation signatures of diverse conditions, including multiple sclerosis, type 1 diabetes and PDAC or chronic pancreatitis (CP). Li et al. (69) utilized the hydroxymethylation profiles of colorectal, gastric, liver, thyroid, and pancreatic cancer and compared them with their adjacent tissues to find definitive signatures that were characteristic of the cancer types. In the last few years, there has been significant progress in the development of biomarkers identifying DNA methylation aberrations that assist in the diagnosis and prognosis of PDAC. **Table 2** below highlights the latest advances in methylated DNA biomarkers that have occurred over the last decade.

**Table 2:** Table showing the latest developments in diagnosis, prognosis, and treatment response prediction of PDAC using DNA methylation biomarkers.

Authors	Year	Target	Method used	Results
Diagnostic biomarker				
Henriksen et al. (70)	2016	28 gene panel based on promoter hypermethylation	Methylation-specific PCR	The diagnostic prediction model showed an AUC of 0.86 in distinguishing PDAC from control with sensitivity of 76% and specificity of 83% respectively
Li et al. (72)	2020	8 biomarker panel(TRIM73, FAM150A, EPB41L3, SIX3, MIR663, MAPT, LOC100128977, and LOC100130148)	Methylated DNA immmunoprecipitation coupled with high-throughput sequencing(MeDIP-seq)	The final prediction model achieved a sensitivity of 97.1% and a specificity of 98.0% on the training cohort, the sensitivity and specificity of the validating cohort was 93.2 and 95.2%, respectively
Majumder et al. (73)	2021	13-methylated DNA marker panel	Target enrichment long-probe quantitative amplified signal method	Test set showed an AUC of 0.84 for biomarker panel alone, 0.87 for CA19-9, and 0.90 for the combined panel. The combined panel was significantly better compared to either(p=0.0382, p=0.0490, respectively).
Miller et al. (74)	2021	ZNF154 methylation	DREAMing analysis	ZNF154 methylation performed better than KRAS with an AUC of 0.85 compared to 0.67 for KRAS.
Wu et al. (75)	2022	56 DNA-methylation based biomarker panel	Reduced representation bisulfite sequencing (RRBS)	The panel achieved an AUC of 0.91 in the validation(sensitivity=84%,specificity=89%) and independent cohort(sensitivity=82%, specificity=88%). The combination of PDACatch and CA 19-9 had an AUC=0.94, which was better than either PDACatch or CA19-9 alone(p<0.001).
Zhao et al. (76)	2024	6 methylation marker panel (KCNA3, PRRX, CCNA1, TRIM58, NR2F1-AS1)	Targeted bisulfite sequencing panel	Diagnostic model with sex methylation markers achieved a sensitivity of 88.7% and specificity of 96.8%. Combining with CA19-9 improved the sensitivity to 95.7% and decreased the specificity to 93.3%.
Kim et al. (77)	2024	7 epigenetic biomarker panel(HOXA9, TWIST, WT1, RPRM, BMP3, NPTX2, BNC1) with KRAS	Bisulfite-free real-time PCR	Plasma cfDNA analyzed using 7-panel biomarker and KRAS exhibited a sensitivity of 90% and specificity of 95% respectively.
Hartwig et al. (78)	2024	50 methylation biomarker based discriminatory panel		Panel distinguished pancreatobiliary cancers from pancreatitis with an AUC of 0.85 and 0.88 in the training(n=45) and validation(n=37) cohorts respectively
Prognostic biomarker				
Heinreksen et al. (71)	2017	28 gene panel based on promoter hypermethylation	Methylation-specific PCR	The prediction model enabled the differentiation of stage I-II from stage III-IV disease (AUC of 0.82 (cut point 0.66; sensitivity 73%, specificity 80%))
Pietrasz et al. (81)	2021	HOXD8, POU4F1	ddPCR	ctDNA was confirmed as an independent prognostic marker for PFS (adjusted hazard ratio (HR) 1.5, CI 95% [1.03-2.18], p = 0.034) and OS (HR 1.62, CI 95% [1.05-2.5], p = 0.029).
Stubbe et al. (82)	2021	hypermethylated SFRP1	deamination followed by PCR	Patients with hypermethylated SFRP1 had a shorter median OS than unmethylated patients(3.2 vs 6.3 months)
Garcia-Ortiz et al. (79)	2023	NPTX2 methylation	ddPCR	Higher circulating NPTX2 methylation levels at diagnosis were associated with poor prognosis and efficiently stratified patients for prediction of overall survival (6.06% cut-off, p = 0.0067)
Koukaki et al. (80)	2024	BRCA1,2 methylation	Methylation-specific PCR	BRCA 1 and 2 methylated status in operable pancreatic cancer had a poorer outcome than non-methylated one (p=0.012, p=0.001, respectively).
Treatment response prediction				
Stubbe et al. (82)	2021	hypermethylated SFRP1	deamination followed by PCR	Gemcitabine-treated patients with hypermethylated SFRP1 had a shorter median overall survival (mOS) (4.4 months) than unmethylated patients (11.6 months).

ddPCR, Digital droplet Polymerase Chain Reaction; mOS, Median overall survival; PFS, Progression-free survival; AUC, Area under curve; ctDNA, Circulating tumor DNA; cfDNA, Circulating-free DNA.

A plethora of studies have been conducted in the last ten years that have furthered our understanding in the role of these DNA methylation biomarkers in the diagnosis of PDAC. One of the earlier studies assessing the role of DNA methylation in the diagnosis of PDAC was conducted by Henriksen et al. (70) in 2016 who employed a 28-gene panel based on promoter hypermethylation in PDAC, CP, and acute pancreatitis patients and created a diagnostic prediction model (age >65, BMP3, RASSF1A, BNC1, MESTv2, TFPI2, APC, SFRP1 and SFRP2) that had an area under the curve of 0.86 with a sensitivity of 76% and specificity of 83% respectively. The authors of that study also studied the 28 gene panel to create a prediction model (SEPT9v2, SST, ALX4, CDKN2B, HIC1, MLH1, NEUROG1, and BNC1) that enabled prognostication by differentiating stage 4 from stage 1-3 disease with an AUC of 0.87 and sensitivity, specificity of 74% and 87% respectively (71). Li et al. (72) performed a genome-wide cfDNA methylation profiling study utilizing immunoprecipitation with high-throughput sequencing and found a total of eight biomarkers that achieved a sensitivity and specificity of 97.1% and 98.0%, respectively, in the training cohort and 93.2% and 95.2% in the validation cohort. Majumder et al. (73) identified a panel of 13 sets of methylated biomarkers that were individually comparable to CA 19-9 and, when combined, performed superiorly compared to either with an AUC of 0.90 (p<0.05). Miller (74) assessed the utility of hypermethylation of the CpG island of ZNF154 as a diagnostic biomarker for multiple cancers, including colon, liver, ovarian, and pancreatic cancer patients. They collected 2711 peripheral blood samples and found that ZNF154 cfDNA methylation outperformed KRAS mutation with an AUC of 0.85 compared to 0.67 for KRAS and were particularly helpful in the diagnosis of early-stage disease (AUC=0.87). Wu et al. (75) developed a targeted methylation sequencing assay called PDACatch utilizing 546 plasma samples from 232 PDAC, 323 healthy controls and 25 patients with CP. Using a 56-marker classifier, they found that this panel differentiated PDAC from healthy controls (HC) and CP with an AUC of 0.91. Importantly, it detected CA19-9 negative PDAC at sensitivities of 75% and 100% in the validation and independent tests, respectively. Utilizing a combination of PDACatch and CA19-9 outperformed either with an AUC of 0.94 (p<0.001). Recently, Zhao et al. (76) identified a six-methylation marker based on liquid biopsy utilizing samples from 262 patients with PDAC and 212 HC and found that the panel achieved high sensitivity and specificity with 88.7% sensitivity for all cases of PDAC and 78% for stage 1 patients. The panel also had a high specificity of 96.8%. Combining the panel with CA19-9 improved the performance further, with a sensitivity of 95.7% and 95.5% for overall cases and stage 1 patients and a specificity of 96.8%. Kim et al. (77) assessed a 7-panel epigenetic biomarker panel developed from 46 patients with PDAC who underwent surgical resection in combination with KRAS and found that the panel showed a sensitivity of 90% and 95% specificity with an overall 93.3% accuracy in discriminating PDAC, which was comparable to 90% showed with CA19-9 and CEA. Hartwig et al. (78) utilized NGS followed by enrichment using methyl-binding domains to compare 25 cases each of PDAC, pancreatitis, controls and seven cases of IPMN and identified a 50 biomarker-based discriminatory panel that showed an AUC of 0.85 and 0.88, respectively, for the training and validation cohort and was also able to distinguish high grade from low-grade IPMN.

Epigenetic biomarkers have also shown great utility in assessing the prognosis of patients with PDAC. Garcia-Ortiz et al. (79) analyzed plasma samples from 44 patients with metastatic PDAC and found that NPTX2 methylation levels at diagnosis were associated with poor prognosis and a cut-off of 6.06% were significant in stratifying patients(p=0.0067) into low-risk (OS-410 days) versus high-risk (OS-187 days). Koukaki et al. (80) tested the applicability of BRCA1 and 2 methylation status in cfDNA as a prognostic biomarker in 55 patients with operable and 50 patients with metastatic pancreatic cancer. They found that in patients with operable pancreatic cancer, a methylated BRCA1 and 2 promoter status conferred a poorer outcome than a non-methylated one (p=0.012, p=0.001, respectively). One of the most important studies in this regard was done by Pietrasz et al. (81) when they prospectively collected 372 plasma samples based on 354 patients targeting two methylated markers (HOXD8 and POU4F1) and found that ctDNA positive for them was an independent prognostic biomarker for PFS (adjusted HR=1.5(1.03-2.18)) and OS(HR=1.62(1.05-2.5)).

While a number of studies have studied potentially useful diagnostic and prognostic biomarkers in DNA methylation for PDAC, a biomarker that can assist with treatment response prediction remains elusive. One such study was performed by Stubbe et al. (82) who exploited secreted frizzled-related protein 1 (SFRP1) hypermethylation as an independent prognostic biomarker for survival and gemcitabine effectiveness in patients with stage 4 PDAC. In a cohort of 40 patients, Stubbe found that hypermethylated SFRP1 was associated with a shorted median OS compared to unmethylated patients (3.2 vs 6.3 months, adjusted HR-3.53(1.85-6.74)). Furthermore, they found that gemcitabine-treated patients with hypermethylated SFRP1 had a shorter median OS of 4.4 months vs 11.6 months in unmethylated patients, thereby predicting response to gemcitabine (HR-3.48(1.39-8.70)). DNA methylation biomarkers represent an interesting prospect that holds great relevance and may become an important part of clinical practice in the following years.

## Circulating tumor cells

CTCs are tumor cells that have separated from the primary tumor and are carried by the bloodstream or the lymphatic system (83). They were first described by Ashworth who had noticed that some cells in the blood of metastatic cancer resembled tumor cells in the primary tumors (84). Although CTCs derive their origin from primary tumours, they behave differently in some ways. The most important feature that distinguishes them from the primary tumour is the epithelial-mesenchymal transformation (EMT) properties that facilitate them to break from the primary tumour, intravasate into the bloodstream, disseminate in clusters of CTC that increase their ability to metastasize and demonstrate stem-cell-like properties (85). Studies have shown the existence of CTC even in the early stages of tumor evolution (86). Due to the EMT property of these cells, it is essential to detect tumor cells not only with epithelial markers available at the origin but also to include mesenchymal markers that improve the sensitivity of their assays. CTC monitoring essentially acts as a monitor for genomic instability and provides additional information with regard to tumor resistance and metastases, serving as a tool for treatment response and precision medicine (83). Molecular and pathological concordance of CTC with the primary tumor has been a topic of debate, with numerous studies showing mixed results.

Traditionally, CTCs were analyzed using FISH and flow cytometry (87). With the advances in molecular biology over the last decade and the advent of single-cell sequencing, it has become possible to conduct extensive genomic, transcriptomic, proteomic and DNA methylation analysis that has broadened our horizon and expanded the utility of CTC. Essentially, CTC technologies can be based on three principles-1) capture and enrichment, 2) detection and identification, and 3) release (88). Capture and enrichment involves interaction between CTC and materials either through physical interactions or antigen-antibody interactions. The second method involves the utilization of ultrasensitive techniques for small quantities of CTC through fluorescence microscopy, spectrophotometry, flow cytometry, electrical impedance, and Raman scattering (88). Released CTC finds utility in genomic, transcriptomic, proteomic analysis and CTC culture. Capture and enrichment based on physical properties often suffer from severe shortcomings, including being inefficient, impure, and lacking specificity. The only advantage is the decreased cost (89). Antigen-antibody-based capture and enrichment utilize EpCAM and vimentin to enrich CTC and negatively enrich CD45 to remove leucocytes (90). This technique has been widely utilized with the CellSearch system based on a similar principle (91). The problem with this system is that CTC are heterogenous with variable EpCAM expression that might lead to inaccurate results (92). New technologies for CTC analysis include nanotechnology-based techniques and microfluidic-based techniques (93, 94). These two techniques transcend the limitations posed by the earlier two techniques but have their own shortcomings. Microfluidic-based cell sorting has not been widely adopted because of the long set-up time, high initial cost, bulky instrumentation, and limited ability to perform single-cell molecular analysis (93). Nanotechnology-based techniques are cost-effective and simple. However, nanoparticle probes’ aggregation and binding can affect the results’ reliability and reproducibility (94).

Numerous studies have explored the role of CTC in PDAC diagnosis, prognosis and treatment prediction. **Table 3** highlighting the studies assessing the diagnostic, prognostic and treatment response prediction properties of CTC has been given below

**Table 3:** Table showing the latest developments in diagnosis, prognosis, and treatment response prediction of PDAC using CTC biomarkers.

Authors	Year	Target	Method used	Results
Diagnostic biomarker				
Liu et al. (96)	2017	Chromosome 8 centromere (CEP8) probe	FISH	Total CTC number had 75.8% sensitivity and 68.7% specificity at a cutoff value of 2. Sensitivity was 53.7% and specificity was 85.4% at a cutoff point of 2 CTC subtypes cells/3.2 mL.
Zhu et al. (106)	2024	Portal venous CTC and peripheral CTC	Microfluidic biopsy	A combination of peripheral and portal CTC data along with CA19-9 results showed to greatly improve the average accuracy of CA19-9-negative PDAC patients from 47.1% with regular CA19-9 tests up to 87.1%.
Prognostic biomarker				
Earl et al. (95)	2015	CTC	CellSearch with CD45-positive cell depletion	CTC detected by CellSearch correlated significantly with OS, 88 days (27-206) for CTC positive samples versus 393 days (284-501) for CTC negative samples. CTC was detected in only 20% of patients, majority of which had metastatic disease.
Okubo et al. (103)	2017	CTC	CellSearch	CTC positivity was associated with a significantly lower OS even after treatments(p=0.045).
Court et al. (102)	2018	CTC	Microfluidic NanoVelcro assay	CTC count correlated with increasing stage (r=0.42, p<0.001). Patient with occult metastatic disease had significantly more CTC than patients with local disease (7 vs 1 CTC, p<0.0001). A cutoff of 3 or more CTC/4mL identified patients with occult metastatic disease preoperatively (AUC 0.82(0.76-0.98)).
Padillo-Ruiz et al. (107)	2021	CTC-portal vs central venous	Immunocytochemistry	Patients with fewer than 185 CTC in portal vein exhibited a longer OS than patients with more than 185 CTC (24.5 vs. 10.0 months; p = 0.018). Similarly, patients with fewer than 15 clusters in portal vein showed a longer OS than patients with more than 15 clusters (19 vs. 10 months; p = 0.004).
Zhao et al. (100)	2021	mesenchymal CTC, hENT-1	CD45 negative enrichment method	Mesenchymal CTC percentage could differentiate locoregional with metastatic disease (0.2 vs 0.345, p=0.0244). It was also an independent prognostic indicator of recurrence-free survival in resected patients(p=0.001). hENT-1 expression in CTC was independent prognostic factor for RFS (p=0.016).
Song et al. (99)	2021	EpCAM, Plectin-1	CellCelector, Whole genome amplification	Patients with detectable EpCAM+ CTC less than one in peripheral blood showed longer overall survival (OS) compared to patients with detectable CTCs more than one (35.5 months vs. 16.0 months).
Xing et al. (104)	2021	CD44+ circulating tumor endothelial cells(CTEC)	Integrated subtraction enrichment and immunostaining-fluorescence *in situ* hybridization (SE-iFISH)	Preoperative CD44+ CTEC was significantly higher in patients with early tumor recurrence(p=0.023)
Cheng et al. (105)	2022	FR+CTC	Ligand-targeted PCR	In the surgical group, median disease-free survival (DFS) for patients with high CTC levels versus low CTC levels (< 14.49 FU/3 ml) was 8.0 versus 26.0 months (P = 0.008).
Nitschke et al. (101)	2022	RARRES1	Microfluidic approach with stable isotope labelling of amino acids in cell culture	CTC positivity (≥3 CTC) at follow up period was significantly associated with short recurrence-free survival (p = 0.002). Furthermore, detection of RARRES1 positive CTCs was indicative of an even earlier relapse after surgery (p = 0.001).
Javed et al. (97)	2022	transitional CTC	Immunofluoroscent staining	Multivariate analysis demostrated that transitional CTC positivity was associated with higher risk of late recurrence (HR:4.7(1.2-18.3))
Javed et al. (98)	2023	transitional CTC	Immunofluoroscent staining	Postoperative transitional CTC were associated with poorer RFS, both in patients with a delay in initiation (12.4 vs 17.9 months, P =0.004) or no administration of adjuvant chemotherapy (3.4 vs not reached, p =0.016).
Treatment response prediction				
Wang et al. (110)	2021	EpCAM positive CTC	Immunomagnetic microspheres and immunofluoroscence	CTC-positive patients with advanced PDAC also had shorter progression-free survival (PFS) after chemotherapy with gemcitabine (P=0.01).
Yu et al. (109)	2022	Gene expression templates for seven chemotherapeutic agents	Collagen adhesion matrix, qPCR	Patients receiving an effective regimen as predicted by the ChemoSensitivity Assay experienced significantly longer mPFS (7.8 months v 4.2 months, HR = 0.35, p = 0.0002) and mOS (21.0 months v 9.7 months, HR = 0.40, p = 0.005), compared with an ineffective regimen.
Lee et al. (111)	2023	CD45, EpCAM, vimentin	Microfabricated filter-based enrichment system followed by immunofluoroscent staining	Total CTC count and vimentin-positive CTC was significantly associated with treatment response during chemotherapy(p=0.024 and 0.017 respectively)
Freed et al. (108)	2023	anti-EpCAM, anti-FAPa	Microfluidic approach	PDAC receiving poly ADP-ribose polymerase inhibitor(PARPi) were evaluated for CTC. Numerical ratio of the number of mesenchymal to epithelial ratio (phi) was used as an indicator of therapy. A decreasing value of phi during treatment was indicative of tumor response to the PARPi. Distinguishing responders from non-responders occured with higher confidence using phi(p=0.0093) compared to CA19-9(p=0.033)

CTC, Circulating tumor cells; PARPi, Poly-ADP ribose polymerase inhibitor; CD45, Cluster of differentiation 45; EpCAM, Epithelial cell adhesion molecule; qPCR, Quantitative-polymerase chain reaction; mPFS, Median progression-free survival; mOS, Median overall survival; HR, Hazard ratio; RFS, Relapse-free survival; DFS, Disease-free survival; CTEC, Circulating tumor endothelial cells; FISH, Fluoroscent *in-situ* hybridization; CEP8, Chromosome 8 centromere.

Earl et al. (95) simultaneously explored the role of CTC and KRAS mutant cfDNA in blood and found that the presence of CTC correlated significantly with OS, 88 days for CTC positive versus 393 days for CTC negative, although they were only detected in 20% of patients. Liu et al. (96) employed FISH to assess the subtype of CTC with chromosome 8 centromere probe in 143 blood samples from PDAC patients and healthy controls and found that both the subtype and total CTC were significantly increased in PDAC patients compared to healthy controls. Total CTC number had 75.8% sensitivity and 68.7% specificity at a cutoff value of 2 cells. 2 CTC subtypes as a cutoff showed a sensitivity of 53.7% and specificity of 85.4%. Javed et al. (97) used transitional CTC to assess recurrence risk one year postoperatively and found that positive transitional CTC was associated with a higher rate of late recurrence (HR=4.7(1.2-18.3)). One-year CTC positivity was associated with a higher rate of recurrence during the second year (OR=13.1(1.6-1953.4)). The same group later also studied the use of transitional CTC to study patients who had a delay in adjuvant therapy by greater than 8 weeks and found that the presence of transitional CTC was associated with a poorer relapse-free survival both in patients with a delay in initiation(p=0.004) as well as no receipt of adjuvant chemotherapy(p=0.016) (98). Song et al. (99) found that postoperative patients with detectable EpCAM-positive CTC of less than one in peripheral blood showed a longer overall survival (35.5 months vs 16.0 months). Zhao et al. (100) differentiated CTC into subtypes based on mesenchymal and human equilibrate nucleoside transporter-1(hENT-1) and found that mesenchymal CTC was detected in 81% of patients and it could differentiate locoregional disease from metastatic disease based on a percentage (p=0.0244). It was also an independent prognostic indicator of recurrence-free survival in resected patients(p=0.001). Nitschke et al. (101) employed RARRES1 expression in CTC using the technique of stable isotope labelling of amino acids in cell culture (SILAC) and concluded that CTC detection on follow-up was significantly associated with short recurrence-free survival(p=0.002), and presence of RARRES1 expression was indicative of an even earlier relapse after surgery(p=0.001). CTC used preoperatively has also been found to have significant implications on prognosis. Court et al. (102) preoperatively assessed CTC in 100 PDAC patients and found that a cut-off of 3 or more CTC in 4 mL correctly identified occult metastatic disease preoperatively (AUC-0.82(0.76-0.98)). Okubo et al. (103) assessed the use of CTC in 65 patients with advanced PDAC (borderline/unresectable) and found that the overall survival rate was significantly lower in patients with positive CTC (p=0.045). This confidence was accentuated further in borderline patients with CTC positivity(p=0.0051). It also served as an independent prognostic factor. Preoperative CD44+ circulating tumor endothelial cells (CTEC) have also been found to be significantly associated with early tumor recurrence (DFS<6 months, p=0.023), as demonstrated by Xing et al. (104). Similarly, folate receptor-positive CTCs have been studied by Cheng et al. (105) who found that there was a significantly decreased median disease-free survival (DFS) for patients with high CTC levels (8 vs 26 months, p=0.008). An area of great interest recently has been the isolation of CTC from portal venous samples, considering the greater proportion of CTC detected closer to the source. Zhu et al. (106) combined peripheral and portal venous CTC with CA 19-9 and found that this combined biomarker panel could improve the accuracy of CA19-9 negative PDAC from 47.1% to 87.1%. Furthermore, portal venous blood samples were found to have twice the number of CTC as peripheral blood (21.4 cells vs 10.9 cells per 5 mL). Padillo-Ruiz et al. (107) also explored this concept by comparing CTC in portal venous blood with a central venous catheter (CVC) and found that while there was no significant correlation observed in CVC analyses, there was a significantly increased OS in patients with less than 185 CTC (24.5 vs 10.0 months, p=0.018) for a sample drawn from the portal vein.

Further studies are required to explore the role of CTC in treatment response prediction in PDAC. One study was conducted by Freed et al. (108) when they assessed the response of PDAC patients on Poly ADP-ribose polymerase inhibitor (PARPi) therapy using a mesenchymal-epithelial CTC ratio(phi). They found that phi(p=0.0093) could differentiate responders and non-responders with higher confidence than CA 19-9(p=0.033). Further, for CA 19-9 non-producers, phi correctly predicted the outcome in 72% of PDAC patients. Yu et al. (109) utilized a chemosensitivity assay to determine an effective regimen based on FOLFOX and gemcitabine-paclitaxel regimen and found that the assay was able to predict an effective regimen with significantly longer PFS (7.8 months vs 4.2 months, HR=0.35, p=0.0002) and overall survival (21 months vs 9.7 months, HR=0.40, p=0.005). Gemcitabine resistance was also studied by Wang et al. (110), who found that CTC-positive patients with advanced PDAC had a shorter PFS after chemotherapy with gemcitabine, which was indicative of treatment resistance(p=0.01). Lee et al. (111) also studied the treatment response prediction ability of CTC and found that CTC count and subtype CTC vimentin positive significantly correlated with treatment response during chemotherapy (p=0.024, p=0.017). Research on CTC has been remarkable and shows great promise in clinical application of PDAC.

## RNA biomarkers

The first instance of circulating free nucleic acids dates back to 1970s when they were isolated from the blood of cancer patients (112). It was initially hypothesized that these circulating free RNAs came from cancer cells. Later, as more research was done the origins of the circulating RNAs were attributed to necrosis and apoptosis of cancer cells and also thought to be actively secreted by the tumors in extracellular vesicles (113). During apoptosis, DNA, RNA, protein, and cellular organelles are packaged in the form of apoptotic bodies and released into circulation. Apoptotic bodies are a component of extracellular membrane vesicles (EMV), which play an important role in phagocytosis and the horizontal transfer of genes (114, 115). The other two parts of EMV include exosomes and microvesicles. Exosomes represent the smallest EMV actively secreted, while microvesicles are produced as a budding of the plasma membrane. Both of them play an important role in intracellular communication (114, 115). Kopreski et al. detected the first extracellular human mRNA in the blood of melanoma patients (116). Following this, multiple other studies found other forms of RNA in the serum or blood (117). RNA are relatively unstable molecules theoretically susceptible to degradation by the abundant ribonucleases in the blood. This is overcome by packaging RNA along with lipids and proteins in the form of vesicles to transport them outside the cell and protect it from outside degradation. The free RNA released into the circulation can be divided into coding RNA and non-coding RNA. Coding RNAs include messenger RNA (mRNA). mRNA are the protein-coding regions that contain the information produced from DNA transcription, which then undergo multiple processes, including 5 methyl capping, splicing, and 3 polyadenylation to produce refined mRNA finally. Analyzing cell-free circulating mRNA can prove an important cancer biomarker tool. Analysis of circulating mRNA provides the benefit of analysis of alternate and splicing variants through which epigenetic transcriptional regulation can be revealed. Human telomerase reverse transcriptase (hTERT) mRNA has been studied in multiple cancers. Telomerase activity and length correlates with cellular senescence (118). hTERT mRNA is upregulated in multiple cancers, including breast cancer, hepatocellular cancer, gastric cancer, and prostate cancer (118). Another notable mRNA that has been extensively studied includes epidermal growth, receptor (EGFR) which is overexpressed in the peripheral blood of patients with pancreatic, non-small cell lung, colon cancer and can be detected using RT-PCR (119, 120). Unfortunately, mRNA molecules typically tend to be between 300 and 4000 base pair in size and due to the long size along with the presence of high RNA activity in the blood, these mRNA molecules are subject to fregmentation, causing significant challenges in identifying biomarkers that have clinical utility. As a result, no reliable mRNA biomarkers have been found to date (121). On the other hand, noncoding RNA (ncRNA) has caused significant excitement in biomarker development. This includes transfer RNA(tRNA), ribosomal RNA(rRNA), micro RNA(miRNA), small interfering RNA(siRNA), piwi-interacting RNA(piRNA), small nuclear RNA(snRNA), small nucleolar RNA(snoRNA), YRNA, circular RNA, pseudogene RNA, and telomerase RNA (122). These ncRNAs vary based on size and function. The size ranges between 18-200 nucleotides long. tRNA and rRNA constitute the housekeeping non-coding RNA (ncRNA). On the other hand, regulatory ncRNA includes miRNA, snRNA and piRNA. The small ncRNA is involved in gene regulation, RNA interference, and spliceosome modification (123). Among these short ncRNA, miRNA have been the most extensively studied for biomarker development for different diseases. These miRNAs are around 20-25 nucleotide long oligonucleotides and have notably been associated with post-transcriptional silencing and expression of genes associated with mRNA (122). They also play an important role in cross-communication between cancer and dendritic cells (124). Besides these functions, they are vital in cell growth, maturation, prognosis, and proliferation, and their dysregulation can cause increased expression of oncogenes present downstream and facilitate cancer development (125). Multiple miRNA targets have also been found to predict the prognosis of malignancy (126).

Another extensively studied RNA includes long noncoding RNA (lnc RNA), which includes circular RNA, pseudogene RNA, and telomerase RNA, ranging from size 400 bp to 4kbp in length. These lncRNA are derived from exosomes and play an important role in metastasis, therapy resistance, angiogenesis, and tumor growth. They have also been known to modify tumor microenvironment, which facilitates cancer development and progression (127). An extensively studied field in lncRNAs include circular RNAs (circRNA). The circRNAs are produced by back splicing, unlike the usual linear RNA slicing process (128). They are considered as co-transcriptional products. Earlier, it was thought that the circRNAs have no role in eukaryotic gene expression. In the last couple of years, the role of circRNAs has been explained in greater detail. Major circRNA biogenesis is mediated by epithelial-mesenchymal transition (EMT) (129). It has also been found to act as miRNA and as latent competitive endogenous RNA molecules that compete with miRNA binding sites (130). circRNAs also consist of both intronic and exonic circRNA. Intronic circRNA can affect inheritance and epigenetics in the cytoplasm. Exonic circRNA can interact with miRNA and mediate miRNA function (129).

The most commonly employed methods for detecting circulating miRNA include quantitative Real-Time PCR (qRT-PCR), hybridization-based technology (Microarray), and high-throughput sequencing (NGS). Out of these three, qRT-PCR is the most widely adopted, largely related to its simple nature, allowing easy use and cost-efficient nature (131). Microarray and NGS, on the other hand, allow a large number of parallel analyses and are useful for genome-wide circulating miRNA profiling and high-throughput detection of circulating miRNA in body fluids. Microarrays are also quite flexible and can be tailored according to needs with a relatively straightforward concept and cheaper cost than NGS (132). The limitations with microarray are the large number of RNA samples needed and the technical variations in additional experimental steps that can affect external validity. As such, it tends to have a lower specificity than qRT-PCR and NGS. NGS is the most promising tool for high-throughput analysis. While qRT-PCR and microarray can only profile known miRNA, NGS quantifies a variety of small RNAs (including unknown ones) with a wide array (133). Deep sequencing facilitates the identification of mutations in miRNA. The high cost and the need for extensive computational infrastructure and bioinformatics support limit its universal adoption (134).

Most of the study surrounding RNA biomarkers in PDAC has been focused on miRNA and lnc RNA. **Table 4** below highlights all the developments in the field of RNA biomarkers in the past few years. One of the largest studies attempted to study the diagnostic value of circulating miRNA in PDAC. It looked at 409 patients with PDAC, 25 patients with chronic pancreatitis and 312 healthy participants. A diagnostic index was developed based on miR–145, 150, 223, and 636. The sensitivity and specificity of the diagnostic miRNA panel combined with CA 19–9 was compared with CA 19–9 alone and found to have significantly increased AUC when compared to CA 19–9 alone (AUC 0.93 vs 0.89) (135). Since then, numerous studies have been conducted over the last decade to find out more potential RNA diagnostic and prognostic biomarkers in PDAC. Kim et al. (136) studied 55 patient samples and analyzed genome-wide expression of serum miRNA in PDAC and biliary tract cancer to identify novel biomarker candidates using sequencing techniques. Three of the highest performing miRNA (miR-744-5p, 409–3p, 128–3p) could distinguish cancer patients from controls with a diagnostic accuracy of 92.7%. An earlier study using miRNA and PDAC was done by Abue et al. (137), who checked the expression of miR-483-3p, and 21 in PDAC. They found that the mean plasma miR-483-3p and 21 were significantly higher in PDAC compared to IPMN and healthy controls. They also found that the presence of miR-21 was associated with lymph node and liver metastasis, suggesting advanced disease. This paved the way for future studies using miRNA biomarkers in PDAC clinical investigations.

**Table 4:** Table showing the latest developments in diagnosis, prognosis, and treatment response prediction of PDAC using RNA biomarkers.

Authors	Year	Target	Method used	Results
Diagnostic biomarkers				
Abue et al. (137)	2014	miR-483-3p,-21	RT-PCR	Mean plasma miR-483-3p significantly higher in PDAC patients compared to IPMN and HC.(AUC=0.754). Mean plasma miR-21 significantly higher compared to IPMN and HC. Plasma miR-21 was also higher in IPMN compared to HC.(AUC=0.790)
Lai et al. (139)	2017	GPC-1, miR-10b,-21, -30c,-106b,-181a, -483,-20,-let7a, and -122a	RT-qPCR	High exosomal miR-10b, -21, -30c, -181a,and low-let7a differentiated PDAC from HC and CP.
Jin et al. (140)	2021	tRF-Pro-AGG-004, -tRF-Leu-CAG-002	qRT-PCR	AUC using the two tsRNA showed an AUC of 0.88 in differentiating PDAC compared to HC
Zhang et al. (141)	2018	LINC00346,00673, 00671,00261,00578, SNHG9	qRT-PCR	LINC00346, 00578, 00673 were highly expressed while LINC00671, 00261, SNHG9 were lowly expressed in serum with AUC0.7073, 0.7837,0.6093, 0.6057, 0.5712, 0.5983 respectively.
Yu et al. (142)	2020	FGA,KRT19, HIST1H2BK, JTIH2,MARCH2, CLDN1,MAL2,TIMP1	RNA sequencing	The d-signature showed high accuracy with AUC 0.950 and 0.936 in the internal and external validation cohort compared to HC and CP. It was superior compared to CA19-9 in comparing PDAC vs CP (AUC=0.931 vs 0.873)
Reese et al. (144)	2020	miR-200b,-200c	qRT-PCR	Combining miR-200b, -200c with CA19-9 from serum exosome and EpCAM-positive exosome increased the accuracy to 97% with a sensitivity of 0.92 and specificity of 1.
Kim et al. (138)	2021	miR-10b,-16,-155, -429,-1290	qRT-PCR	A combination panel of miR-10b,-155,-429,-1290 along with CA19-9 had the highest sensitivity for diagnosis (AUC-0.964, sens-100, specificity-80%)
Guo et al. (145)	2021	miR-95-3p,-26b-5p	RNA sequencing	Ratio of miR-95-3p divided by miR-26b-5p in combination with CA19-9 had a sensitivity of 96. 5% and specificity of 96.4%.
Liu et al. (146)	2012	miR-16,-196a	real-time PCR	A combination of CA 19-9 with miR-16,-196ahad an AUC of 0.979 for discriminating PDAC from non-PDAC with a sensitivity of 92% and specificity of 95.6%.
Li et al. (147)	2013	miR-1290	real-time PCR	miR-1290 yielded an AUC of 0.96 for PDAC compared to HC with a sensitivity of 88% and specificity of 84%
Wei et al. (148)	2020	miR-1290,-1246	qRT-PCR	Combining miR-1290,-1246 with CA 19-9 had an AUC of 0.99 with sensitivity 96.7%and specificity of 97.5% against HC.
Qu et al. (149)	2017	miR-21-5p	qRT-PCR	Circulating miR-21-5p showed an AUC 0.78 compared to HC with sensitivity of 0.77 and specificity of 0.80.
Wang et al. (150)	2020	miR-133a	qRT-PCR	miR-133a demonstrated an AUC of 0.893 with a sensitivity of 90.6% and specificity of 87.2%.
Nakamura et al. (151)	2022	13 marker(5 circulating and 8 exosomoal microRNA)	qRT-PCR	The 13 marker microRNA panel showed an AUC=0.93 in the validation cohort for distinguishing PDAC from HC. The AUC was same for early-stage PDAC. In combination with CA19-9, the AUC increased to 0.99.
Yu et al. (152)	2020	miR-25	qRT-PCR	miR-25 with CA19-9 had an AUC=0.985 for distinguishing PDAC from HC with a sensitivity of 97.5% and specificity of 90.11%.
Chen et al. (143)	2022	miR-451a	qRT-PCR	Exosomal miR-451a showed an AUC of 0.896 and 0.855 for distinguishing HC and benign pancreatic disease respectively.
Liu et al. (153)	2024	miR-200	qRT-PCR	Combined expression of the miR-200 family showed an AUC of 0.823. In an independent validation cohort, application of this model showed a sensitivity, specificity and AUC of 100%, 88%, and 0.97 respectively, for diagnosing PDAC.
He et al. (154)	2024	LINC01268, LINC02802, AC124854.1, and AL132657.1	Whole-transcriptome sequencing, qRT-PCR	Exosomal LINC01268, LINC02802, AC124854.1, and AL132657.1 had an AUC of 0.8421, 0.6544, 0.7190, and 0.6321 respectively and the AUC of the four exosomal lncRNAs increased to 0.8476, with a sensitivity of 0.72 and specificity of 0.89.
Prognostic biomarkers				
Abue et al. (137)	2014	miR-483-3p,-21	RT-PCR	miR-21 expression associated with advanced stage and metastases to lymph node and liver. OS in high vs low expression(3 months vs 13.8 months)
Zhang et al. (141)	2018	LINC00346,00673, 00671, 00261,00578, SNHG9	qRT-PCR	High expression of LINC00346, 00578, 00673 or low expression of LINC00671, 00261, SNHG9 had significantly lower survival percent.
Reese et al. (144)	2020	miR-200b,-200c	qRT-PCR	Multivariate analysis showed miR-200b in EpCAM-positive serum exosomes as an independent prognostic factor (OS 9 months(high) vs 18 months(low))
Guo et al. (145)	2021	miR-335-5p,-340-5p	RNA sequencing	Ratio of miR-335-5p divided by -340-5p greater than 0.15 had a worse OS (median OS-205 vs 413 days)
Treatment response prediction				
Chen et al. (143)	2022	miR-451a	qRT-PCR	miR-451a levels decreased in PDAC patients following treatment. Serum levels were remarkably increased in those who had recurrence while it was unchanged in those in remission.

RT-PCR, Real-time polymerase chain reaction; IPMN, Intraductal papillary mucinous neoplasm; HC, Healthy controls; miR-Micro-RNA; CP, Chronic pancreatitis; tsRNA- tRNA-derived small RNAs; AUC-Area under curve; lncRNA-Long noncoding RNA; OS-Overall survival.

Another study conducted by Kim et al. (138) captured extracellular vesicles using magnetic beads and identified 5 miRNAs, including miR–10b, 16, 155, 429, and 1290 markedly elevated in PDAC. When combined with CA 19–9, the diagnostic panel reported a sensitivity of 100%, specificity of 80% and an AUC of 0.964. The biggest challenge in PDAC has been the ability to differentiate chronic pancreatitis from PDAC. Lai et al. (139) used miRNA signature and exosomal glypican to find a panel which can distinguish chronic pancreatitis from PDAC. They found that exosomal miRNA (high exosomal miR-10b, -21, -30c, -181a, low miR-let7a) was superior to exosomal glypican and CA19-9 in differentiating PDAC from chronic pancreatitis. Jin et al. (140) described another interesting study in which they did small RNA sequencing of 30 patients with PDAC and 30 healthy controls and found that more variations were detected among tRNA-derived small RNA (tsRNA) than miRNA. They used this finding to validate findings in 24 separated PDAC patients and healthy controls using the most significantly variable tsRNA and found two potential candidate tsRNA(tRF-Pro-AGG-004, tRF-Leu-CAG-002) with an AUC=0.88 in differentiating PDAC from healthy controls.

A substantial effort has also been made to explore the role of lncRNA in PDAC. Zhang et al. (141) studied the expression levels of six lncRNA in serum and tissues of patients with PDAC (LINC00346, LINC00578, LINC00673, LINC00671, LINC00261, and SNHG9). LINC00346, LINC00578, and LINC00673 were highly expressed, whereas LINC00671, LINC00261, and SNHG9 were expressed at lower levels when compared with healthy controls. Their expression levels also correlated with the clinical stage of the disease. Besides, those with higher and lower expression levels of the corresponding lncRNA also had a significantly lower OS. Yu et al. (142) explored another avenue when they conducted a case-control study with 501 participants (284 with PDAC, 100 with chronic pancreatitis (CP), and 117 healthy subjects). They performed an EV long RNA sequencing (evLR) and utilized a machine learning algorithm to develop a diagnostic signature of eight unique evLR(FGA, KRT19, HIST1H2BK, ITIH2, MARCH2, CLDN1, MAL2 and TIMP1). This panel of evLR showed a high diagnostic accuracy of distinguishing PDAC from healthy controls with an AUC of 0.960, 0.950 and 0.936 in the training, internal validation and external validation cohort, respectively. The signature also performed well in distinguishing early (stage 1 and 2) PDAC with an AUC of 0.949. When compared with CA19-9 in distinguishing PDAC from CP, the signature was significantly superior (AUC= 0.931 vs 0.873, p=0.028).

Research in this area needs to focus more on utilizing RNA biomarkers for treatment response prediction. Chen et al. (143) utilized serum miR-451 as a tool for treatment response. They found that miR-451 decreased to nearly undetectable levels after resection and treatment. After treatment, miR-451 also proved valuable as a surveillance tool to detect recurrence. A rebound increase in miR-451 during the surveillance period highly correlated with recurrence, while levels of patients in remission did not change considerably. miR-451 could also be used as a diagnostic biomarker and showed an AUC of 0.896 and 0.855 in distinguishing PDAC from healthy controls and benign pancreatic diseases, respectively. RNA biomarkers can be a game-changer in the management of PDAC. The challenges hindering the translation of miRNA and lncRNA into clinical practice include primarily the associated cost and technical expertise. Future research needs to focus on more biomarkers to guide treatment decisions and find ways to implement them in day-to-day clinical practice and lowering the associated cost.

## Protein biomarkers

Proteins dynamically interact with each other having numerous intermolecular interactions and undergoing numerous posttranslational modifications. Their roles in modulation of molecular processes and pathways makes protein biomarkers relevant in tumorigenesis and progression (155). Liquid biopsy for proteins involves numerous technologies: Mass spectrometry (MS), antibody/antigen arrays, aptamer-based assays, proximity extension assays (PEA) and reverse phase protein arrays (RPPA) (156). This is in addition to traditional techniques such as ELISA, chemiluminescence and radioimmunoassay, which are cheaper and usually have a single target (157). MS-based proteomics is widely used these days in conjugation with liquid chromatography (LC) for liquid biopsy screening (158).MS technology allows large-scale untargeted proteomic and targeted analysis rapidly. Several studies have focused on protein profiling using the matrix-assisted laser desorption/ionisation time-of-flight (MALDI-TOF) and surface-enhanced laser desorption/ionisation time-of-flight (SELDI-TOF) (159, 160). MS, however, requires significant optimization and expertise and is often time-consuming. They may also be affected by abundant proteins, and many strategies have been implemented to deplete them, including ultrafiltration, solid phase and organic solvent extraction, and serum or plasma fractionation (161). While traditional ELISA is a singleplex assay, many contemporary multiplex methods have been developed that measure many target proteins over a wide range without the need for any depletion. These assays are dependent on specific antibodies or modified aptamers (162). Affinity-based protein profiling assays depend on antibodies or aptamers that recognize specific epitopes, without cross-reactivity to other proteins (162). There have been relatively few studies examining the performance of these assays, and it remains critical for proteomics-related liquid biopsy. While excellent as an exploratory tool, the scalability, inter-assay variation and costs of antibody/antigen arrays are often the rate-limiting steps in designing a study pipeline. Aptamers are short single-stranded DNA, RNA, or peptides that, upon folding into specific tertiary structures, bind to cognate protein targets in native states with high affinity and specificity (163). Aptamers have a higher affinity and specificity than antibodies, and can be readily synthesized and selected *in vitro* with low variation, allowing a cost-efficient way to make it scalable (163). It is a topic of great scope although aptamers available to the research communities are still limited compared to antibodies. In PEA multiple antibody pairs for proteins of interest are pooled and labelled with complementary DNA oligo sequences to allow high-fidelity hybridization (164). This happens only when true antibody pairs are in proximity to target proteins. The dsDNA is then PCR-amplified, allowing measurement of relative concentration of target proteins. PEA has outperformed LC-MS methods, presenting wider dynamic ranges with high accuracy and reproducibility (164, 165). The limitation is the tedious library preparation and NGS requirement which can contribute to biases and intra-inter-experimental variations when high sample size throughput is in place (164). Another technique that has captured attention is RPPA. In RPPA, fully denatured protein lysates are immobilized onto solid substrates, and this process can be repeated to allow any number of targets to be interrogated (166–168). RPPA allows robust parallel large sample profiling ranging from hundreds to a few thousand samples. RPPA is especially useful in blood cancer and other liquid biopsies due to its ability to track intra-cellular proteins (166). Limitations with RPPA involve sophisticated experimental workflow, prolonged experimental process (with more proteomics), slow turnaround time, and the validation of RPPA-usable antibodies

The quantitative measurement of proteome poses a greater challenge than assessing the genome. Around one million different proteins are present in the human body, through various combinations of epigenetic regulations, differential RNA splicing and post-translational modifications. In contrast, there are only 22,000-25,000 coding genes within the human genome (169). Proteins are in a constant state of flux, with rapid changes in abundance and dynamically modifying as a response to all kinds of environmental stimuli. The genome, in contrast, is relatively stable with slow rate of change.

Historically, there has been a great deal of emphasis on the utility of protein biomarkers for diagnosing and treating PDAC. **Table 5** below highlights all the major developments in the field of protein biomarkers in the past few years.The most well-known serological biomarker in PDAC is carbohydrate antigen 19-9(CA19-9). CA19-9 has only been reported to be elevated in 80% of all patients with PDAC. It has also traditionally been utilized to monitor disease progress or treatment responsiveness. There can also be false positives with benign conditions such as chronic pancreatitis, biliary obstruction and cholangitis (170, 171). A meta-analysis conducted in 2018 to assess the diagnostic value of CA19-9 in PDAC had concluded the sensitivity of CA19-9 as 0.80 in the diagnosis of PDAC and a specificity of 0.75 with an area under the curve (AUC) of 0.84 (172). Another important biomarker discovered in the last decade is CA242. A meta-analysis comprising 21 studies and 3497 participants was conducted in 2015 to check the utility of cancer-antigen 242(CA242) in conjunction with CA19-9 and CEA in diagnosing PDAC. A sensitivity as high as 90% was achieved by combining CA19-9 with CA242 (173). Another study utilized a biomarker panel of CA19-9, serum periostin and CA242, which was able to distinguish early stage PDAC from controls with an AUC of 0.98 and benign conditions with an AUC of 0.90 (174).

**Table 5:** Table showing the latest developments in diagnosis, prognosis, and treatment response prediction of PDAC using protein biomarkers.

Authors	Year	Target	Method used	Results
Diagnostic biomarkers				
Cheng et al. (185)	2020	FR+CTC, CA 19-9	LT-PCR, chemiluminescence	The combination of FR+CTC with CA19-9 showed high sensitivity 97.8% and specificity 83.3%.
Velstra et al. (175)	2015	m/z peak values 2084, 2178, 2770, 2899, 3096, 8760, 8939	Mass spectrometry	The discriminating profile had a sensitivity of 74% and specificity of 91% for pancreatic cancer with an AUC=0.90.
Park et al. (176)	2017	LRG-1, TTR, CA 19-9	Multiple reaction monitoring, mass spectrometry	AUC=0.931 for the panel in discriminating PDAC from HC. It differentiated PDAC from benign pancreatic diseases (AUC=0.892).
Nie et al. (177)	2014	α-1-antichymotrypsin (AACT), thrombospondin-1(THBS1), and haptoglobin(HPT)	Mass spectrometry	The panel showed high diagnostic accuracy in distinguishing PDAC with OJ(AUC=0.92) or without OJ (AUC=0.95).
Aronsson et al. (186)	2018	CA 19-9, IL-17E, B7.1, and DR6	Glycosylation antibody array	The combined panel gave an AUC of 0.988 for discriminating stage 1 PDAC and healthy controls with 100% sensitivity and 90% specificity.
Jenkinson et al. (187)	2016	Thrombospondin	LC-MS	A combination of TSP-1 and CA19-9 demonstrated an AUC of 0.85 for PDAC compared to CP and HC.
Han et al. (178)	2015	Dickkopf-1	ELISA	ROC curve showed that DKK-1 was significantly better than CA19-9 in differentiating PDAC from controls.
Ren et al. (180)	2014	Interleukin-11	ELISA	IL-11p showed the highest diagnostic accuracy for PDAC (AUC-0.901, sensitivity-97.7%, specificity-70%).
Wang et al. (183)	2014	MIC-1/GDF15	ELISA	MIC-1 performed better than CA19-9 in distinguishing early-stage PDAC with a higher sensitivity (62.5% vs 25%).
Mellby et al. (188)	2018	29 biomarker signature	Microarray	Samples derived from patients with stage 1 and 2 PDAC showed an AUC of 0.96 compared to control.
Kaur et al. (189)	2017	MU5AC, CA 19-9	ELISA, RIA	Combining MU5AC with CA19-9 improved the sensitivity and specificity to 83% with an AUC of 0.91 to distinguish benign and CP controls.
Lee et al. (190)	2014	Human complement factor B	Immunoprecipitation coupled to MS	The combination of CFB and CA19-9 improved the sensitivity and specificity to 90.1 and 97.2%, respectively.
Guo et al. (191)	2016	Dysbindin	Mass spectrometry	At the optimal cutoff, dysbindin had an AUC=0.849 with a sensitivity of 81.9% and a specificity of 84.7%.
Wu et al. (192)	2019	PROZ, TNFRSF6B	LC-MS	The combination of two markers with CA 19-9 demonstrated an AUC of 0.919 with a sensitivity of 75.6% and specificity of 95%.
Papapanagiotou et al. (193)	2018	Osteonectin	ELISA	Osteonectin showed an AUC of 0.856 with a sensitivity of 84.6% and a specificity of 87.5%.
Balasenthil et al. (194)	2017	TFPI, TNC-FN-IIIC	ELISA	The panel showed an AUC of 0.86 in discriminating PDAC from HC with a sensitivity of 0.81 and specificity of 0.8
Honda et al. (195)	2012	apoAII-ATQ/AT	ELISA	A combination of CA19-9 and apoAII-ATQ/AT showed an AUC of 0.879 for PDAC of 0.879 for PDAC compared to HC.
Hogendorf et al. (196)	2018	GDF-15, IL-17, IL-23	ELISA	GDF-15 had a sensitivity of 73.8% and a specificity of 76.19%
Mohamed et al. (197)	2014	ADH, MIC-1	ELISA	A combination of two markers with CA19-9 showed an AUC of 0.89 for stage 1 and 2 PDAC from HC and 0.92 for stage 3 and 4 PDAC from HC.
Wei et al. (198)	2020	Exo-Epha2	ELISA	A combination of Exo-Epha2 with CA19-9 CA242 showed an AUC of 0.97 for distinguishing PDAC from HC with a sensitivity of 90% and a specificity of 97.6%, and an AUC of 0.93 with a sensitivity and specificity of 88.5 and 96.6% for benign diseases.
Song et al. (199)	2019	OPN, MIA, CECAM-1, MIC-1, SPON1, HSP27	Multiplex immunoassay	The four best biomarkers to separate PDAC from HC were MIC-1(0.97), CA 19-9(0.93), CECAM-1(0.91), and OPN (0.90)
Melo et al. (200)	2015	Glypican-1	Immunogold TEM	GPC1 circulating exosomes showed a perfect AUC with a sensitivity and specificity of 100%.
Lewis et al. (201)	2014	Glypican-1, CD-63	Immunoassay	PDAC patients could be identified from HC with a sensitivity and specificity of 99% and 82%, respectively.
Xiao et al. (179)	2020	GPC1, CD82, CA 19-9	Immunoassay, flow cytometry	The combined panel showed an AUC of 0.942 to distinguish HC from PDAC
Byeon et al. (202)	2024	Polymeric Immunoglobulin Receptor(PIGR), von Wilebrand Factor(vWF), fibrinogen, SAA1, THBS1, CRP	High resolution-mass spectrometry	PIGR and vWF showed a high ability to diagnose early-stage (Stage 1 and 2) PDAC patients(AUC=0.8926), which improved after the introduction of CA19-9 to the panel(AUC=0.9798).
Yablecovitch et al. (203)	2024	MMP-7, SDC1	ELISA	The AUC for MMP-7 and SDC-1 in PDAC versus controls was 0.90 and 0.78, respectively. A combination of the two and CA19-9 showed an AUC of 1.0(p<0.001).
Prognostic biomarkers				
Han et al. (178)	2015	Dickkopf-1	ELISA	The overall median survival was 9 months for a group with higher DKK1 compared to 15 months for a group with lower DKK1.
Ren et al. (180)	2014	Interleukin-11	ELISA	IL-11p correlated with OS (high vs low IL-11p OS-10 months vs 4 months).
Kim et al. (181)	2019	human complement factor B	Immunoprecipitation	There was a significant difference in DFS between the two groups (36.9 vs 13.9 months, respectively).
Uemara et al. (204)	2024	*Amaranthus caudatus* agglutinin(ACA) positive EV	Liquid chromatography	The OS and RFS of patients with higher ACA-positive EVs were significantly higher than those with lower ACA-positive EVs (26.1 months vs not reached and 11.9 vs 38.6 months respectively). ACA-positive EV elevation in postoperative serum was an independent prognostic factor for poor OS(HR=3.891, p=0.023) and RFS(HR=2.650, p=0.024).
Treatment response prediction				
Tsutsumi et al. (182)	2012	CA 19-9, DUPAN-2, Span-1, CEA	ECLIA, EIA, IRMA	A combination of CA19-9 and Span-1 could predict treatment failure in 72%(35/49) during gemcitabine therapy.
Wang et al. (183)	2014	MIC-1/GDF15	ELISA	One month after potentially curative resection, MIC1 levels decreased to levels similar to benign tumors, and levels increased again after tumor recurrence.
Peng et al. (184)	2019	PZ, SHBG, vWF, AZGP-1	LC-MS	A combination of these four biomarkers demonstrated the best response in distinguishing good responders from limited responders

LT-PCR, Ligand targeted polymerase chain reaction; FT+CTC, Folate receptor positive circulating tumor cells; m/z, Mass-to-charge ratio; AUC, Area under curve; TSP, Thrombospondin; DKK-1-Dickkopf-1, LC-MS, Liquid chromatography-mass spectrometry; ELISA, Enzyme-linked immunosorbent assay; IL, Interleukin; RIA, Radioimmunoassay; CFB, Complement factor B.

Velstra et al. (175) performed one of the earliest biomarker analyses utilizing mass spectrometry when they utilized mass spectrometry peaks to identify a unique biomarker signature that could be utilized in PDAC. The discriminating profile demonstrated a sensitivity of 74% and a specificity of 91%in distinguishing PDAC from controls with an AUC of 0.90. Another exciting study utilizing mass spectrometry was done by Park et al. (176)when they identified a unique panel of protein biomarkers comprising leucine-rich alpha-2 glycoprotein (LRG1), transthyretin (TTR), and CA19-9 from a potential 1000 biomarkers from 134 clinical samples using mass spectrometry. The triple biomarker panel outperformed CA19-9 with an AUC of 0.931 compared to 0.826 for CA19-9 in diagnosing PDAC compared to controls. The superiority was even more prominent in differentiating PDAC from benign pancreatic disease and other cancers. Nie et al. (177) studied a combination of alpha-1-anti chymotrypsin, thrombospondin-1, and haptoglobin, which outperformed CA19-9 in distinguishing PDAC from normal controls, diabetes, cyst, and chronic pancreatitis. A combination of these markers was capable of distinguishing PDAC from other conditions with (AUC=0.92) or without obstructive jaundice (AUC=0.95). Dickkopf-1 (DKK1) was studied by Han et al. (178) as an alternative to CA 19-9 when they obtained serological levels of both the markers using ELISA and followed up the patient for two years to find that DKK1 was significantly better in distinguishing patients with PDAC from controls (AUC=0.919 vs 0.853). The study also found DKK1 as a useful prognostic biomarker, with the OS being 9 months for samples with higher DKK1 levels compared to 15 months for patients with lower DKK1 levels. Xiao et al. (179) have also explored the use of flow cytometry and immunoassay in the diagnosis of PDAC using a combined panel comprising glypican-1, CD82, and serum CA19-9. The combined panel effectively distinguished PDAC from healthy population (AUC-0.942). Ren et al. (180) have also explored the use of interleukin-11 as both a potential diagnostic and prognostic biomarker. On comparing the serum levels of IL-11 in 44 patients with PDAC with healthy controls, median baseline levels of IL-11 levels of patients with PDAC were significantly higher than those of healthy controls (p<0.001). IL-11 as a biomarker showed a sensitivity and specificity of 97.7% and 70.0% respectively with an AUC of 0.901 in distinguishing PDAC from healthy controls. Patients with distant metastases were found to have lower median levels of IL-11 and demonstrated a correlation with overall survival (lower than median IL-11 had 4 months compared to 10 months for those with higher median IL-11 levels). Another prognostic biomarker which has been studied in the past includes complement factor B(CFB). Stratifying patients on the cutoff value for CFB, Kim et al. (181) found that there was a significant difference in the DFS and OS for patients with low versus high CFB(DFS- 36.9 vs 13.9 months, p:0.007; OS-49.7 vs 29.0 months, p:0.048).

Numerous studies have also explored the use of protein biomarkers as a treatment response and prediction tool. One of the earliest studies to explore this was by Tsutsumi et al. (182), who explored the utility of tumor markers such as CEA, CA19-9, duke pancreatic mono-clonal antigen type 2(DUPAN-2) and s-pancreas antigen-1 (Span-1) to determine if it can assist in earlier confirmation of treatment failure. Their study yielded that a combination of CA 19-9 and Span-1 could facilitate a more accurate determination of treatment failure than CA 19-9, finding a failure in 72% of patients (p=0.004). Wang et al. (183) utilized macrophage inhibitory cytokine 1(MIC-1) as a treatment response monitoring tool and found that MIC-1 levels significantly decreased a month after curative resection in PDAC, and the levels rebounded whenever there was a tumour relapse that had occurred. As a diagnostic biomarker as well, MIC-1 performed better than CA19-9 showing a higher sensitivity (65.8% vs 53.3%). It was also more effective in distinguishing early-stage PDAC from normal serum compared to CA19-9(62.5% vs 25%). Peng et al. (184) utilized MS to identify a unique biomarker panel of PDAC patients with good response versus poor responders and found that a composite biomarker panel of PZ, SHBG, vWF, and CA 19-9 could segregate good responders from limited responders effectively.

While protein biomarkers still remain one of the most widely studied topics in PDAC, the approach suffers from a few shortcomings. Irrelevant clinical questions and underpowered designs falling short of statistical significance are major issues hindering protein biomarkers’ development. Other important factors that prevent the implementation of protein biomarkers involve the technology standards expected and needed for biomarker discovery and the lack of reproducibility in real-world settings. Despite all the above challenges, proteins represent an interesting avenue with vast potential that can be tapped into. CA19-9 represents a great success story in terms of PDAC, and it is hoped that more such biomarkers can be developed that find implementation and use in daily practice.

## Blood-based multiomic biomarkers

The shortcomings in each set of biomarkers have spurred an interest in utilizing a combination of DNA, RNA and protein biomarkers to determine the diagnosis, prognosis and treatment response in PDAC. An interesting study was conducted by Cohen et al. (205)utilizing a combination of ctDNA (KRAS mutation), CEA, CA19-9, hepatocyte growth factor (HGF), osteopontin (OPN) through polymerase chain reaction and immunoassay and found that the combination biomarker panel increased the sensitivity to 64% and the specificity to 99.5%. Another groundbreaking study exploring the use of miRNA and protein biomarker panel was conducted by Madhavan et al. (206), who utilized a combination of flow cytometry and qRT-PCR to optimise a panel of miRNA and protein biomarkers that could detect PDAC with a sensitivity of 100% and specificity of 80%. One of the latest studies in this approach was conducted by Chen et al. (207) when they used PCR and chemiluminescence to use a combination of circulating tumour cells and CA19-9 as a diagnostic panel for PDAC and found that the combination demonstrated high sensitivity and specificity of 97.8% and 83.3% respectively.

Utilizing a combination approach while improving the detection rates and sensitivity sometimes adds the shortcomings of technical expertise and overhead expenses which come along with the addition of techniques. This can at present be only practiced in higher centers which are equipped with facilities and money to bear these expenses. **Table 6** shows a summary of combination strategies utilizing DNA, RNA, protein biomarker studied in the management of PDAC.

**Table 6:** Table showing strategies utilizing a combination of DNA, RNA, protein biomarker.

Authors	Year	Target	Method used	Results
Diagnostic biomarkers				
Cohen et al. (205)	2017	ctDNA(KRAS)+CEA+CA 19-9+OPN+HGF	PCR and immunoassay	The combination of KRAS with protein panel increased the sensitivity to 64% and specificity to 99.5%
Madhavan et al. (206)	2015	miRNA(-1246. 4644, 3976,-4306), anti CD9, CD63, CD151	qRT-PCR, flow cytometry	Concomitant evaluation of protein and exosome marker improved sensitivity to 100% and specificity to 80% for PDAC with all other groups
Chen et al. (207)	2022	CTC+ CA199	FISH, chemiluminescence	Integrating CA199 and CTC showed an AUC of 0.95 in distinguishing PDAC from control

ctDNA, Circulating tumor DNA; CEA, Carcinoembryonic antigen; qRT-PCR, Quantitative real-time PCR; CTC, Circulating tumor cells; FISH, Fluorescent *in-situ* hybridization.

## Therapeutic applications and targeted therapy

Advanced technologies such as NGS have also facilitated the development of many targeted therapies based on a specific cell receptor/target/mutation. PDAC, based on the mutation status detected in NGS, can be divided into four subgroups: stable, locally rearranged, scattered, and unstable (208). Combining genomic and transcriptomic data with the relevant proteomic status allows a much more pragmatic classification of PDAC with therapeutic targets. Numerous oncogenes have been explored and evaluated for potential targets. The most commonly mutated oncogene in PDAC is KRAS. Ongoing phase 1 and 2 clinical trials are assessing the utility of MRTX849, a small molecule that selectively modifies mutant cysteine in KRAS G12C (209). First-generation EGFR inhibitors tried for this purpose, such as erlotinib and gefitinib, failed to show any substantial benefit (median HR=0.94, 95% confidence interval 0.76-1.15, p=0.26) (210). The lack of response was thought to be secondary to the resistance caused by the non-EGFR members of the ERBB family. To overcome this issue, irreversible tyrosine kinase inhibitors such as afatinib and neratinib were developed. Ongoing clinical trials (NCT02451553) assess afatinib’s efficacy in PDAC (211). Another EGFR inhibitor, nimotuzumab, was evaluated in locally advanced or metastatic PDAC in a phase 2 trial where the median OS was significantly improved with its use (OS=8.6 vs 6.0 months, HR=0.69, p=0.03) (212). Efforts have also been made to combine EGFR inhibitors with other pharmaceuticals to bypass the equivocal benefit seen with the use of a single agent. A phase 2 trial (NCT01222689) utilizing the combination of erlotinib plus selumetinib in metastatic PDAC showed modest antitumor activity with a median OS of 7.3 months (213).

Proteins downstream of KRAS, including RAF/PI3K/AKT/mTOR, have also been studied as potential targets of interest. MEK inhibitors, including selumetinib and trametinib, were tried in advanced PDAC but failed to yield significant results (selumetinib HR=1.03,p=0.92; trametinib HR=0.98, p=0.453) (214, 215). BKM120, a PI3K inhibitor combined with selumetinib, has shown promising results in murine models but is still nascent and requires further investigations (216).

The results have been slightly better when looking at targets beyond KRAS. A subsection of patients with KRAS harbour other mutations, such as NTRK and NRG1 (125). TRK inhibitors have recently entered the foray and include drugs including larocectinib and entrectinib found to have efficacy in PDAC harbouring these mutations (larocectinib response rate was 79%, and the entrectinib response rate was 57%) (217, 218).

TP53 is one of the most commonly inactivated tumor suppressors in PDAC, with the incidence found to be around 70% in individuals with PDAC (127). COTI-2, a TP53 reactivator drug, is currently being studied (NCT02433626) to evaluate its efficacy and utility in improving the prognosis of patients with TP53 mutant PDAC (219). Another potential target currently being studied involves CDK4/6. Inspired by the success of ribociclib and palbociclib in metastatic breast cancer and liposarcoma, they were tested in murine models of PDAC, where they demonstrated great promise, and they are currently also being evaluated in clinical trials (NCT02501902) (220). Another success story that derives its roots from breast cancer includes PARP inhibitors such as olaparib. A recent prospective phase 3 trial (NCT02184195) performed to evaluate the efficacy of olaparib in patients with mutant BRCA metastatic PDAC found that the PFS was increased significantly in the olaparib group (PFS 7.4 months versus 3.8 months, HR=0.53, p=0.004) (221).

Targeted therapy adds to the arsenal of options available at our disposal for treatment of PDAC. The success in targeted therapy especially in lung cancers and the rapid progression in techniques of liquid biopsy creates optimism with respect to improved treatment outcomes with targeted therapy in PDAC.

## Future directions and conclusion

**Figure 1:**
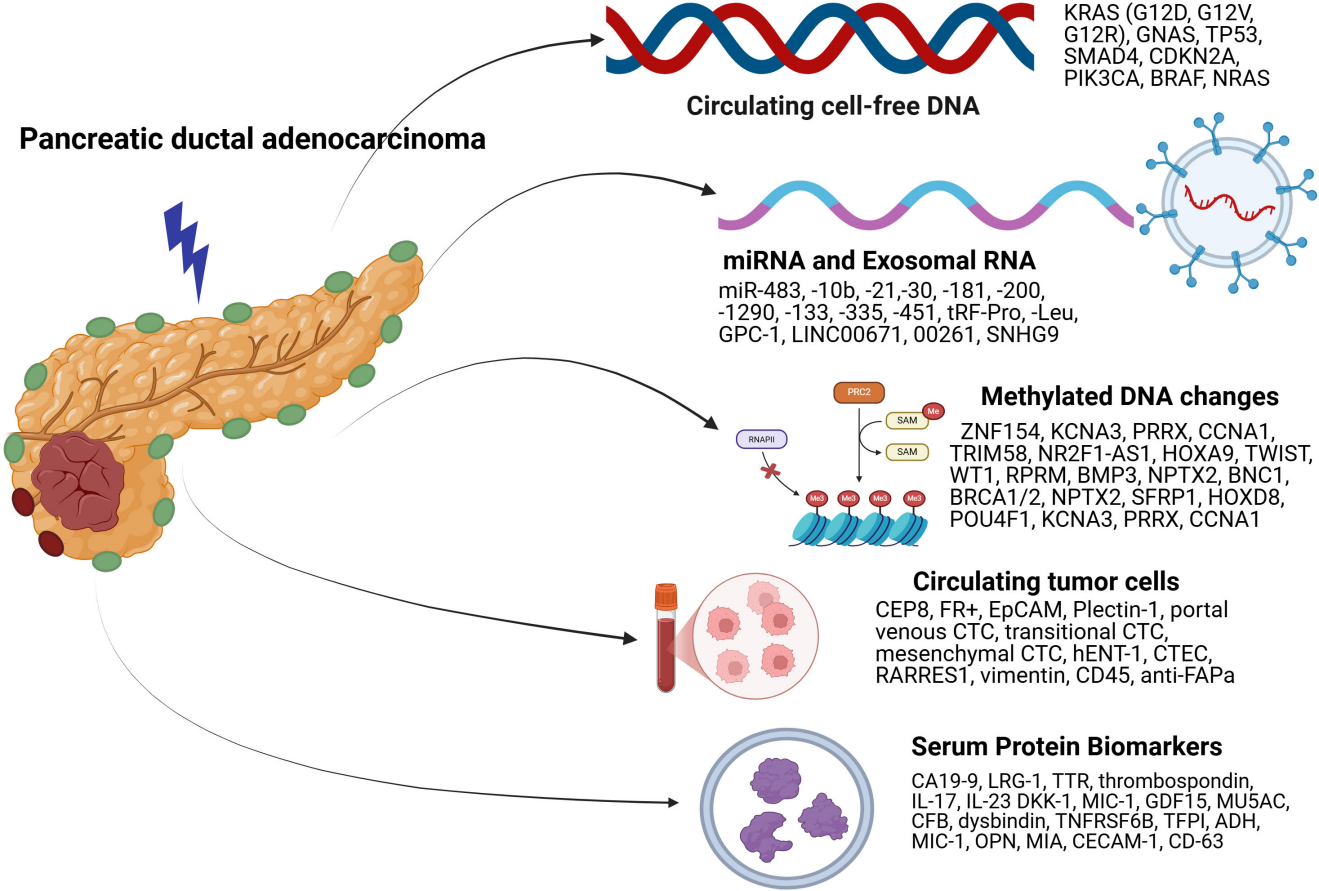
Illustration summarizing latest developments in serum DNA, RNA, CTC, DNA methylation, and protein biomarkers.

While numerous advances have been made in terms of biomarkers facilitating the diagnosis and prognosis of PDAC, few biomarkers have been explored which allow treatment response prediction. More effort needs to be focused on this area since they fit much more seamlessly into clinical practice and allow a greater understanding and clarity about the natural course of the disease. It also allows the oncologist to be prepared for failure and think about other options for treatment and allows patients to be better prepared about the consequences of the cancer. Utilizing a combination of genomics, transcriptomics and proteomics allows a greater sensitivity and certainty and gives greater discriminatory power to distinguish benign pancreatic diseases from PDAC. Efforts should be made to decrease the expenses associated with it so that the economic costs can be accommodated within the total treatment cost that is entailed with the diagnosis of PDAC. Biomarker discovery in PDAC holds great promise and utilizing it with imaging evidence and clinical suspicion may hold the solution to the conundrum of early diagnosis of PDAC allowing complete curative treatment of the disease. **Figure 1** demonstrates an illustration highlighting the latest developments in DNA, RNA, and protein biomarkers.
